# Antithymocyte Globulin Plus Post-Transplant Cyclophosphamide Combination as an Effective Strategy for Graft-versus-Host Disease Prevention in Haploidentical Peripheral Blood Stem Cell Transplantation for Children with High-Risk Malignancies

**DOI:** 10.3390/ph15111423

**Published:** 2022-11-17

**Authors:** Kang-Hsi Wu, Te-Fu Weng, Ju-Pi Li, Yu-Hua Chao

**Affiliations:** 1 Department of Pediatrics, Chung Shan Medical University Hospital, Taichung 402, Taiwan; 2 Department of Pediatrics, School of Medicine, Chung Shan Medical University, Taichung 402, Taiwan; 3 Department of Pathology, School of Medicine, Chung Shan Medical University, Taichung 402, Taiwan; 4 Department of Clinical Pathology, Chung Shan Medical University Hospital, Taichung 402, Taiwan

**Keywords:** antithymocyte globulin, children, graft-versus-host disease, haploidentical transplantation, post-transplant cyclophosphamide

## Abstract

Haploidentical hematopoietic stem cell transplantation using post-transplant cyclophosphamide (PTCy) for graft-versus-host disease (GVHD) prophylaxis has emerged as a valid alternative transplant strategy for patients lacking a suitable HLA-matched related donor. The high risk of severe GVHD remains the major clinical challenge in this setting. The addition of antithymocyte globulin (ATG) in PTCy-based regimens for GVHD reduction in haploidentical hematopoietic stem cell transplantation is rational and was reported in adult series. However, its feasibility is unknown in pediatric patients. Here, we firstly describe our experience of 15 consecutive children with high-risk malignancies receiving haploidentical peripheral blood stem cell transplantation using ATG plus PTCy for GVHD prophylaxis. Only three patients developed grade 1–2 acute GVHD, limited to skin. No grade 3–4 acute GVHD and chronic GVHD were observed. Viral reactivations were frequently seen but manageable. Six patients relapsed, as the main cause of death in our series. None died from events related to GVHD. Our data suggest that ATG plus PTCy is an effective strategy for GVHD prevention in haploidentical peripheral blood stem cell transplantation and is feasible in children with high-risk malignancies.

## 1. Introduction

Allogeneic hematopoietic stem cell transplantation (HSCT) is a potentially curative therapeutic option for children with high-risk malignant diseases; but only 30% of candidates have an HLA-matched related donor [1]. Haploidentical HSCT has been extensively used as an alternative transplant strategy. The advantages are the immediate availability of a donor, the possibility of performing cell therapy after transplantation, and potentially greater graft-versus-tumor effects. However, the high transplant-related mortality from graft-versus-host disease (GVHD) remains the major clinical challenge of haploidentical HSCT.

Administration of post-transplant cyclophosphamide (PTCy) was found to attenuate graft-versus-host reactions in mice receiving major histocompatibility complex-incompatible cells [2]. The Johns Hopkins group pioneered the use of PTCy to prevent GVHD in haploidentical bone marrow transplantation [3]. Since then, PTCy has become the standard prophylaxis of GVHD in haploidentical HSCT. However, the risk of GVHD remains high, and patients with severe GVHD usually have a dismal prognosis. Therefore, a variety of modifications have been proposed to improve GVHD probability and severity in haploidentical HSCT with PTCy, including ex vivo graft manipulation of T cell-depletion, the use of reduced-intensity conditioning regimens, and the addition of agents with immunosuppressive activities [4].

Antithymocyte globulin (ATG) can deplete donor and recipient T cells and, thus, reduce the risk of graft rejection and GVHD. It has been widely used for GVHD prophylaxis in matched unrelated donor HSCT [5,6]. The efficacy of adding ATG in PTCy-based regimens on GVHD reduction in haploidentical HSCT were documented in adult patients [7]. However, its feasibility remains unknown in children receiving haploidentical HSCT with PTCy. Herein, we present our results of 15 consecutive transplantations for children with high-risk malignant diseases who received haploidentical peripheral blood stem cell transplantation (PBSCT) using ATG plus PTCy for GVHD prevention. To our knowledge, this is the first report regarding haploidentical HSCT with the combination of ATG and PTCy as GVHD prophylaxis in children.

## 2. Results

### 2.1. Patient Characteristics

From January 2017 to December 2021, a total of 15 pediatric patients with malignant diseases undergoing haploidentical PBSCT in our institution were enrolled in this study. Since 2017, ATG plus PTCy combination has been used as the regular GVHD prophylaxis in all pediatric patients receiving haploidentical PBSCT in our hospital. Clinical features of the 15 patients are summarized in Table 1.

There were eight males and seven females. All patients were less than 18 years old at haploidentical PBSCT, and the median age was 9.2 years (range, 2.1–16.7). All patients had high-risk malignant diseases. The majority of patients (12 of 15) received haploidentical PBSCT for their relapsed/refractory leukemia, seven with acute myeloid leukemia (AML), four with acute lymphoblastic leukemia, and one with chronic myeloid leukemia (CML). Among the eleven patients with acute leukemia, seven could achieve molecular complete remission with undetected minimal residual disease before transplantation. However, three patients even failed to achieve morphologic remission at the time of haploidentical PBSCT. One (Patient 12) with CML had sustained MR2 and failed to achieve a major molecular remission. Three patients received haploidentical PBSCT for their relapsed solid tumors. One (Patient 15) with advanced rhabdomyosarcoma received chemotherapy after the first relapse but still had residual tumor in her nasopharynx before the haploidentical PBSCT. Three patients had a prior HSCT. One (Patient 7) with AML, who received PBSCT from a matched unrelated donor after the first relapse, suffered from a relapse again and was suggested to receive haploidentical PBSCT. Both patients with neuroblastoma (Patient 13 and 14) had prior autologous PBSCT and relapsed. There was no detected residual tumor before haploidentical PBSCT in these two patients.

All donors were haploidentical relatives. The father was the donor in most cases (*n* = 11), followed by a sibling (*n* = 3). Six of the donors were ABO-mismatched. All patients and their donors were positive for cytomegalovirus (CMV) immunoglobulin G in their serum, indicating CMV seropositivity.

### 2.2. Engraftment and Chimerism

Transplant-related information is summarized in Table 2. The conditioning regimen was individualized based on the underlying disease, age, and clinical conditions of the patient. Peripheral CD34+ cells were infused unmanipulated, and the median dose was 7.54 × 10^6^/kg (range, 6.02–9.04). Neutrophil engraftment occurred at a median of 14 days, ranging from 10 to 20 days. All of the 15 patients achieved complete donor chimerism by day +60 measured in the whole blood compartment. No graft failure was observed in our series of patients.

### 2.3. Acute and Chronic GVHD

In addition to cyclosporine (CsA) and mycophenolate mofetil (MMF), ATG plus PTCy combination was used for GVHD prophylaxis in all of the patients. To maintain a good balance between supporting graft-versus-tumor effects and suppressing graft-versus-host reactions, the total dose of ATG was adjusted, three patients with 4.5 mg kg^−1^, six with 2.5 mg kg^−1^, and six with 2.0 mg kg^−1^. According to the modified Glucksberg criteria, the incidence of acute GVHD was 20.0% (three of fifteen patients). In these patients, acute GVHD was grade 1–2 only, limited to skin. They were treated with topical steroids, and all responded well to treatment. None of the patients had gut and liver involvement. No patients in our series experienced severe acute GVHD (grade 3–4). In terms of the National Institutes of Health criteria, none developed chronic GVHD.

### 2.4. Post-Transplant Complications

Viral reactivations were frequently observed in our series. Thirteen of the fifteen patients (86.7%) experienced CMV reactivation, which was defined as an elevation of >200 copies/mL of CMV DNA in the plasma. Intravenous ganciclovir was preemptively administered in these patients. Despite preemptive treatment initiated when there were evidences of CMV reactivation, one (Patient 2) developed CMV disease of retinitis. He was successfully treated with concurrent use of intravenous CMV immunoglobulin and ganciclovir. Five patients (33.3%) demonstrated Epstein–Barr virus (EBV) reactivation and were treated with rituximab. Among them, one (Patient 12) had EBV-related post-transplant lymphoproliferative disease (PTLD) proven by histopathology of the enlarged lymph node, and successfully managed by the R-CHOP regimen. Two patients (13.3%) developed grade 4 hemorrhage cystitis secondary to BK virus (BKV), presenting significant urinary retention by macroscopic hematuria with blood clots. Low-dose cidofovir was given intravenously along with hydration support symptomatic treatment. Both patients had clinical response to treatment and none developed acute renal injury. Totally, multiple viral reactivations were observed in five patients.

Eight patients (53.3%) had probable invasive aspergillosis documented by imaging studies and elevated aspergillus galactomannan antigen levels. All of these patients received preemptive therapy with voriconazole. All patients, except one (Patient 8), were successfully treated. Blood stream infection was documented in 4 patients: *Escherichia coli* in one, *Staphylococcus epidermidis* in two, and *Pseudomonas aeruginosa* in one. All of the patients were responding well to antibiotic treatment, without contributing to mortality. Veno-occlusive disease of the liver, as defined by the modified Seattle criteria [8], were observed in two patients (Patient 11 and 13) within 30 days after transplantation. Both patients were successfully treated with defibrotide.

### 2.5. Outcomes

Follow-up duration ranged from 6 months to 4.2 years. Eight patients (53.3%) remain alive, and seven patients died. The median follow-up of alive patients was 20 months (range, 6 months to 4.2 years) and of deceased patients, 7.0 months (range, 2.5–16 months). No patient in our series died from events related to GVHD.

Seven of the twelve patients (58.3%) with leukemia are alive, with the median follow-up time of 11 months (range, 6 months to 4.2 years). Disease recurrence was the most common cause of mortality in these patients. Four of the five deceased patients died from relapse, and one (Patient 8) died of invasive fungal infection of aspergillosis. Disease status at transplantation had a major impact on the survival outcome. Five of the seven patients with acute leukemia (71.4%) receiving haploidentical PBSCT in molecular complete remission are alive. On the contrary, all of the three patients who did not achieve morphologic remission at transplantation died.

Although allogeneic HSCT is not a routine clinical practice for pediatric patients with high risk, recurrent, or refractory solid tumors, graft-versus-tumor effects after allogeneic HSCT may be a great benefit to these patients who usually have an extremely poor prognosis with current therapeutic regimens [9,10]. In our series, three patients with advanced solid tumors suffered from recurrence and were suggested to receive haploidentical PBSCT. One (Patient 14) with relapsed metastatic neuroblastoma remains alive (>4.2 years) without any evidence of active disease after successful haploidentical PBSCT. The other one (Patient 13) with stage IV neuroblastoma had multiple recurrence in the primary lesion (left adrenal gland), lymph nodes and bone marrow after haploidentical PBSCT. Multiple pulmonary metastases were found soon after haploidentical PBSCT in Patient 15, who had relapsed rhabdomyosarcoma with residual nasopharyngeal tumor before transplantation. Both patients with recurrence diseases after haploidentical PBSCT respond poor to subsequent treatment and died.

## 3. Discussion

Here, we report our experience with implementation of ATG plus PTCy as GVHD prophylaxis in our haploidentical PBSCT program for pediatric high-risk malignancies. The outcome data of a cohort of 15 consecutive children were analyzed in this study. Of note, the incidence of acute and chronic GVHD were very low. Only three patients developed mild acute GVHD, limited to skin. No grade 3–4 acute GVHD and chronic GVHD were observed in the series of patients. Viral reactivations were frequently seen but manageable. Thirteen patients (86.7%) with CMV reactivation, five (33.3%) with EBV reactivation, and two (13.3%) with BKV-related grade 4 hemorrhage cystitis. There was no transplantation-related mortality related to GVHD. For the first time, our data showed that ATG plus PTCy combination appeared to be an effective strategy for GVHD prevention in haploidentical PBSCT in children. Viral reactivations should be monitored closely and treated promptly.

As an alternative source of stem cells for allogeneic HSCT, an eligible HLA-haploidentical related donor could be identified rapidly in nearly all patients. The major problem of haploidentical HSCT has been associated with a significantly high mortality rate from severe GVHD and graft rejection [11], which are manifestations of the intense bi-directional alloreactivity due to HLA mismatch. PTCy as an important composition of the regimen to overcome GVHD and graft rejection in haploidentical HSCT was firstly proposed by Luznik et al. [3]. Since then, PTCy has been extensively used for patients receiving HLA-mismatched allogeneic HSCT and become the standard prophylaxis of GVHD in haploidentical HSCT. Nevertheless, data focusing on haploidentical HSCT with PTCy in children are relatively limited, all regarding small cohorts of patients. Using myeloablative conditioning regimens in haploidentical HSCT with PTCy, there was a very high incidence of acute GVHD in children, ranging from 35 to 43% [12,13]. Of importance, a significant proportion of the patients died from infections related to severe GVHD. Even with the use of reduced-intensity conditioning regimens along with PTCy to reduce the risk of GVHD in children with haploidentical HSCT, the incidence of acute GVHD remained high, ranging from 19 to 43% [10,14,15,16]. It is worth noting that grade 3-4 acute GVHD was observed in 13 to 25% of the patients. Additionally, the incidence of chronic GVHD ranged from 7 to 40%. Therefore, strategies to improve GVHD probability and severity in this setting are needed.

ATG has been extensively used for GVHD prophylaxis in matched unrelated donor HSCT with beneficial effects on decreasing acute and chronic GVHD [5,6]. It appears rational to achieve an extra benefit in terms of GVHD reduction with addition of ATG to the backbone of PTCy in haploidentical HSCT. The impact of adding ATG as a part of the pretransplant conditioning in haploidentical HSCT with PTCy was evaluated by El-Cheikh et al. in a series of adult patients with hematologic malignancies [7]. Compared to those with PTCy only, patients receiving ATG and PTCy had lower rates of acute GVHD and GVHD-related mortality, without compromise of outcomes. In the present study, ATG plus PTCy combination was used for GVHD prophylaxis in a series of 15 consecutive children with malignant diseases receiving haploidentical PBSCT. Instead of bone marrow, granulocyte colony-stimulating factor (GCSF)-mobilized peripheral blood stem cells (PBSCs) have become the major stem cell source worldwide, though they have a greater risk of GVHD. Despite using PBSCs as the cell source in our series, the incidence of acute GVHD was very low; only three patients with mild acute GVHD limited to skin. No patients had grade 3–4 acute GVHD, and none developed chronic GVHD, either. This is the first report regarding haploidentical HSCT with ATG plus PTCy for GVHD prophylaxis in children, showing the best result of GVHD prevention in the literature.

The total dose of ATG was adjusted to trend lower in our series. In the initial three patients, rabbit ATG at 4.5 mg kg^−1^ was added to the backbone of PTCy. Each of the three patients experienced viral reactivations, and two had invasive aspergillosis. On the contrary, only one developed mild acute GVHD. Giving serious consideration to that our patients were at very high risk, graft-versus-tumor effects to some degree may be beneficial to disease control. Therefore, the total dose of ATG was adjusted from 4.5 to 2.5 mg kg^−1^ and then to 2.0 mg kg^−1^. It is interesting to note that results of GVHD prophylaxis remained excellent even with the low dose of 2.0 mg kg^−1^. Accordingly, we speculated that there might still be room for a decline of the ATG dose in this setting. Further studies with larger cohorts of patients are warranted to determine the optimal ATG dose in children receiving haploidentical PBSCT with PTCy.

The excellent results of GVHD prophylaxis came as quite a surprise to us, and we tried to find possible underlying factors in this regimen which might also contribute to decreasing the risk of GVHD. First, the combined intestinal decontamination medication of oral metronidazole and ciprofloxacin was used in our series. The influence of intestinal bacterial decontamination on the occurrence of acute GVHD was documented by Beelen et al., showing that ineffective growth suppression of intestinal anaerobic bacteria was independently associated with a 1.7-fold higher risk of acute GVHD [17]. Metronidazole predominantly covers anaerobic bacteria. Compared to ciprofloxacin alone, combined use of metronidazole and ciprofloxacin as antibacterial prophylaxis in adult patients receiving HSCT improved anaerobic decontamination efficacy and reduced the incidence of grade 2–4 acute GVHD (18% vs. 52%) [18]. The efficacy of enteral metronidazole in GVHD prophylaxis were also demonstrated in children following allogeneic HSCT [19]. Second, high-dose methylprednisolone was used as premedication of ATG in our patients. Cross-reactions of ATG with lymphocytes and macrophages can induce an intense release of cytokines and produce symptoms such as chills, fever, and skin rashes. Premedication with high-dose prednisolone was found to decrease cytokine release and alleviate discomforts in patients receiving allogeneic HSCT [20]. As known, steroids exhibit immunosuppressive potential. Despite its short-term use, the influence of high-dose methylprednisolone on the immune system may be more durable than expected.

In our series, viral reactivations were frequently seen. CMV reactivation was detected in 86.7% patients. As initiation of preemptive therapy at lower CMV viral loads was associated with shorter time for resolution of viremia [21], a relatively low cut-off value of 200 copies/mL CMV DNA in the plasma was used to define CMV reactivation in our study and all patients with positive results received preemptive ganciclovir treatment. Although one developed CMV retinitis, he was successfully treated with CMV immunoglobulin and ganciclovir. As PTLD is one of the most severe complications in allogeneic HSCT and is strongly correlated with EBV DNAemia [22], preemptive treatment has been recommended in patients at high risk of developing PTLD [23]. In our series, five patients demonstrated EBV reactivation (33.3%) and were treated with rituximab. One developed biopsy-proven EBV PTLD and successfully rescued by the R-CHOP regimen. Additionally, two patients had severe BKV hemorrhage cystitis. Based on quantitative viral load measurement by polymerase chain reactions (PCR), viral reactivation can be closely monitored and preemptive therapy can be properly initiated. Along with the advance of approaches to viral diseases, considerable improvements in outcomes can be achieved in this setting. While the incidence was high in our series, none died from viral reactivations.

It is quite difficult to evaluate whether addition of ATG to the backbone of PTCy for GVHD prophylaxis in haploidentical HSCT can exert beneficial influence on the survival outcome in children with malignancies. In studies of using PTCy only, the reported survival rates ranged greatly, between 13.3% and 64.3% [10,12,13,14,15,16]. Many factors can influence the chances of survival in these patients. The disease status itself is of most importance: hematologic malignancies or solid tumors, with or without specific genetic mutations, refractory or relapsed diseases, achieving remission at transplantation or not. Nevertheless, the overall survival rate of 53.3% in the present study was comparable to the rates of about 50% in most previous reports. It may be more objective to compare the survival outcomes in children with non-malignant diseases, because GVHD accounts for the vast majority of mortality and there is no concern of disease relapse in these patients. In the present study, disease recurrence was the major cause of death and the high relapse rate can be partially explained by their high-risk and advanced-stage diseases. Although a variety of novel strategies for prevention and treatment of relapse after HSCT have been proposed [24,25,26], future efforts on improving relapse probability in this setting are needed. Of importance, the incidence of GVHD was very low and none died from events related to GVHD in our series. Even those with relapse after transplantation, they had several months to enjoy a high quality of life without severe GVHD until their primary disease progressed. On the other hand, ATG plus PTCy combination for GVHD prophylaxis may be promising for children with non-malignant diseases who need to receive haploidentical PBSCT, because GVHD is the major cause of mortality in this setting.

## 4. Materials and Methods

### 4.1. Patients

This study was approved by the Institutional Review Board of the Chung Shan Medical University Hospital (CS2-22105). Clinical records of pediatric patients who underwent haploidentical PBSCT for their high-risk malignant diseases between January 2017 and December 2021 at our hospital were retrospectively reviewed. A total of 15 consecutive patients under 18 years of age were enrolled for analysis.

### 4.2. Donor Selection and Stem Cell Source

The current practice in our institute for patients needing to receive HSCT therapy but in the absence of a suitable HLA-matched related donor is to use haploidentical donors. The haploidentical donors were first-degree relatives or siblings who were HLA-haploidentical based on high resolution molecular typing at HLA-A, B, Cw, DRB1, and DQB1. Serum CMV immunoglobulin G was determined in all patients and their donors, and a positive result indicated CMV seropositivity. PBSCs were collected from GCSF-mobilized peripheral blood. Since the population of cells bearing CD34 antigens are thought to be responsible for engraftment after transplantation, graft assessments by flow cytometric quantitation of CD34+ cells have been regularly used [27]. The target dose of CD34+ cells was 5–9 × 10^6^/kg, and the CD34+ cells were infused unmanipulated. 

### 4.3. Conditioning Regimens and GVHD Prophylaxis

The conditioning regimen was individualized based on the underlying disease, age, and clinical conditions of the patient. For GVHD prophylaxis, all patients received PTCy (50 mg/kg/day) on days +3 and +4 along with rabbit ATG (Thymoglobuline^®^; Genzyme, Marcy-ľÉtoile, France) on days −3, −2, and −1. Intravenous mesna (Uromitexan^®^; Baxter Oncology GmbH, Halle, Germany) was administered to prevent cyclophosphamide-related hemorrhagic cystitis. The total dose of ATG was adjusted, from 4.5 to 2.0 mg kg^−1^. Methylprednisolone (3–4 mg kg^−1^, maximum 250 mg; Solu-Medrol^®^; Pfizer, Puurs, Belgium) was given before and 6 h after the start of ATG to alleviate ATG-related symptoms, such as high fever and chillness [20]. Patients also received CsA (3 mg kg^−1^/day; Sandimmun^®^; Novartis, Stein, Switzerland) and MMF (15 mg kg^−1^ thrice a day; CellCept^®^; Roche, Segrate, Italy), both starting on day +5. MMF was discontinued on day +35. The target trough levels of CsA were between 150 and 300 ng mL^−1^. CsA tapering was started around day +60, and it was discontinued on day +90 if no acute GVHD. 

### 4.4. Supportive Care

All patients were treated in protective isolation rooms provided with high-efficiency particle air filters, and supportive care was performed according to our institutional guidelines. Prophylactic antibiotics consisted of ciprofloxacin (Seforce^®^; Nang Kuang, Tainan, Taiwan), metronidazole (Fresenius Kabi Deutschland GmbH, Friedberg, Germany), micafungin (Micamine^®^; Astellas, Toyama, Japan), trimethoprim-sulfamethoxazole (Morcasin^®^; Sinphar, Yilan, Taiwan), and acyclovir (zovirax^®^; GlaxoSmithKline, Parma, Italy). GCSF treatment of 10 μg/kg/day was started on day +5 and continued until neutrophil engraftment. Laboratory evaluation, including a complete blood count and chemistry panel, hepatic and renal function tests, and urinary analysis, was performed twice a week. Intravenous immunoglobulin (Privigen^®^; CSL Behring AG, Bern, Switzerland) was given whenever the immunoglobulin G level was <400 mg/dL. Cefepime (Cefim^®^; Yung Shin, Taichung, Taiwan) alone or in combination with teicoplanin (Targocid^®^; Sanofi, Anagni, Italy) was given as empirical therapy for febrile neutropenia. Quantitative PCR for CMV and EBV were tested every week to detect viral reactivation. The cut-off value for test positivity was >200 copies of CMV DNA per milliliter of plasma. Any positive result above that level was considered CMV reactivation and preemptive treatment with intravenous ganciclovir (Cymevene^®^; F. Hoffmann-La Roche, Basel, Switzerland) was initiated. The cut-off value for test positivity was >600 copies/mL of EBV DNA in the plasma. Any positive result over that level was considered EBV reactivation, and intravenous rituximab (MabThera^®^; F. Hoffmann-La Roche, Basel, Switzerland) was given. PCR screening for BKV in the blood and urine was done weekly. The aspergillus galactomannan antigen assay (Platelia^TM^ Aspergillus Ag; Bio-Rad, Marnes-la-Coquette, France) was performed twice a week. Chimerism was assessed by short tandem repeat analysis at day +60, and full donor chimerism was defined as >95% donor cells. 

## 5. Conclusions

The main limitations of this study were the small cohort of patients and the short follow-up. Nevertheless, it is important to highlight that the ATG plus PTCy strategy as GVHD prophylaxis in haploidentical PBSCT resulted in a low incidence of GVHD with reasonable outcomes in children with malignancies. Viral reactivations were frequent seen but usually manageable; closely monitoring of viremia and prompt intervention are required. Future prospective multicenter clinical trials of large cohorts of patients are needed to validate the results and optimize the protocol.

## Figures and Tables

**Table 1:** Patient characteristics and donor information.

Patient No.	Sex	Age (Years)	ABO Blood Type	Diagnosis	Disease Status at Haploidentical PBSCT	Prior HSCT	Donor Information	
							Donor	ABO Blood Type
1	M	3.9	O	AML, M7	CR1, MRD < 0.01%	No	Father	A
2	M	12.4	B	AML, M0	MRD: 0.12%	No	Father	A
3	M	8.6	B	AML with *FLT3*-ITD	CR1, MRD < 0.01%	No	Father	B
4	F	13.9	O	AML with *FLT3*-TKD	Not morphologic remission	No	Mother	O
5	F	2.1	O	AML with *RUNX1* mutation	CR1, MRD < 0.01%	No	Father	A
6	M	15.2	A	AML with *FUS-ERG*, relapse	CR2, MRD < 0.01%	No	Father	A
7	F	15.4	O	AML, 2nd relapse	Not morphologic remission	MUD PBSCT	Sister	O
8	F	4.8	A	Early T-cell precursor ALL	Not morphologic remission	No	Father	A
9	M	12.1	A	T-cell ALL	CR1, MRD < 0.01%	No	Father	O
10	F	6.2	A	ALL, relapse	CR2, MRD < 0.01%	No	Father	O
11	M	16.7	O	ALL, relapse	CR2, MRD < 0.01%	No	Brother	O
12	F	12.2	O	CML, poor response to TKIs	MR2 *	No	Sister	O
13	M	5.4	A	Stage IV neuroblastoma, relapse	No residual tumor detected	Autologous PBSCT	Father	O
14	M	6.8	O	Stage IV neuroblastoma, relapse	No residual tumor detected	Autologous PBSCT	Father	O
15	F	9.2	O	Stage IV rhabdomyosarcoma, relapse	Residual tumor in nasopharynx	No	Father	O

ALL: acute lymphoblastic leukemia; AML: acute myeloid leukemia; CML: chronic myeloid leukemia; CR: complete remission; F: female; *FLT3*-ITD: internal tandem duplication mutation of the *FLT3* gene; *FLT3*-TKD: tyrosine kinase domain mutation of the *FLT3* gene; HSCT: hematopoietic stem cell transplantation; M: male; MR: molecular response; MRD: minimal residual disease; MUD: matched unrelated donor; PBSCT: peripheral blood stem cell transplantation; TKI: tyrosine kinase inhibitor. * MR2: sustained *BCR-ABL* levels between 0.1% and 1% and failure to achieve major molecular remission.

**Table 2:** Transplant-related information and outcomes.

Patient No.	Conditioning Regimen	ATG Dose (mg kg^−1^)	Infused CD34+ Cells (×10^6^ kg^−1^)	Days to ANC > 0.5 × 10^9^/L	Acute GVHD (Grade)			Asp	Viral Reactivation			Survival after Haploidentical PBSCT	Cause of Death
					Skin	Gut	Liver		CMV	EBV	BKV Cystitis		
1	Bu/Flu/Mel ^#^	2	8.69	19	-	0	0	-	Yes	-	-	Alive (6+ mo)	-
2	Flu/TBI *	2	7.13	14	Yes	0	0	Yes	Yes	-	-	Alive (2.5+ y)	-
3	Clo/Bu/Flu/TBI ^§^	2.5	7.54	14	-	0	0	Yes	Yes	-	-	Dead (7.7 mo)	Relapse
4	Flu/TBI *	2.5	6.41	20	-	0	0	Yes	Yes	-	-	Dead (7.0 mo)	Relapse
5	Bu/Flu/Mel ^#^	2	9.04	13	Yes	0	0	-	-	-	-	Alive (9+ mo)	-
6	Bu/Flu/Mel ^#^	2	6.02	17	-	0	0	-	Yes	Yes	-	Alive (11+ mo)	-
7	Flu/TBI *	2.5	6.54	10	-	0	0	-	Yes	-	-	Dead (4.2 mo)	Relapse
8	Bu/Flu/TT ^†^	2	8.09	18	Yes	0	0	Yes	Yes	-	-	Dead (3.7 mo)	IFI
9	Flu/TBI *	2	8.99	15	Yes	0	0	-	Yes	Yes	-	Alive (1.0+ y)	-
10	Bu/Flu/TT ^†^	2.5	8.79	17	-	0	0	Yes	Yes	Yes	Yes	Alive (2.4+ y)	-
11	Flu/TBI *	2.5	6.81	13	Yes	0	0	Yes	Yes	-	-	Dead (2.5 mo)	Relapse
12	Flu/TBI *	4.5	7.36	14	-	0	0	-	-	Yes	-	Alive (4.2+ y)	-
13	Flu/TBI *	2.5	7.91	15	Yes	0	0	Yes	Yes	-	Yes	Dead (16 mo)	Relapse
14	Flu/TBI *	4.5	8.21	14	Yes	0	0	Yes	Yes	Yes	-	Alive (4.2+ y)	-
15	Flu/TBI *	4.5	7.12	12	Yes	0	0	-	Yes	-	-	Dead (8.3 mo)	Relapse

ANC: absolute neutrophil count; Asp: aspergillosis; ATG: antithymocyte globulin; CMV: cytomegalovirus; EBV: Epstein-Barr virus; GVHD: graft-versus-host disease; IFI: invasive fungal infection; mo: months; PBSCT: peripheral blood stem cell transplantation; y: years. (+) indicates patient who remain alive; (-) indicates no. * Flu/TBI: fludarabine 30 mg m^−2^/day on days −7 to −5; total body irradiation with a total dose of 12 Gy. ^§^ Clo/Bu/Flu/TBI: clofarabine 30 mg m^−2^/day on days −13 to −9; busulfan 3.2 mg kg^−1^/day on days −5 to −3; fludarabine 40 mg m^−2^/day on days −5 to −2; total body irradiation 200 cGy on day −1. ^#^ Bu/Flu/Mel: busulfan 1.2 mg kg^−1^/day on days −6 to −4; fludarabine 30 mg m^−2^/day on days −6 to −2; melphalan 140 mg m^−2^ on day −1. ^†^ Bu/Flu/TT: busulfan 1.2 mg kg^−1^/day on days −7 to −4; fludarabine 30 mg m^−2^/day on days −7 to −3; thiotepa 10 mg kg^−1^/day divided into two doses on day −2.

## Data Availability

Data is contained within the article.
